# The effect of Epstein–Barr virus viremia on the progression to severe COVID-19

**DOI:** 10.1097/MD.0000000000029027

**Published:** 2022-05-13

**Authors:** Jae Hyoung Im, Chung Hyun Nahm, Young Soo Je, Jin-Soo Lee, Ji Hyeon Baek, Hea Yoon Kwon, Moon-Hyun Chung, Ji-Hun Jang, Jung Soo Kim, Jun Hyeok Lim, Mi Hwa Park

**Affiliations:** a Division of Infectious Diseases, Department of Internal Medicine, Inha University College of Medicine, Incheon, Republic of Korea; b Department of Laboratory Medicine, Inha University School of Medicine, Incheon, Republic of Korea; c Department of Laboratory Medicine, Seoul Clinical Laboratories (SCL), Yongin, Republic of Korea; d Department of Internal Medicine, Seogwipo Medical Center, Jeju-do, Republic of Korea; e Department of Hospital Medicine, Inha University School of Medicine, Incheon, Republic of Korea; f Division of Critical Care Medicine, Department of Internal Medicine, Inha University College of Medicine, Incheon, Republic of Korea; g Division of Pulmonology, Department of Internal Medicine, Inha University Hospital, Inha University School of Medicine, Incheon, Republic of Korea.

**Keywords:** cellular immunity, coronavirus 2019, critical illness, Epstein–Barr virus infection, latent infection

## Abstract

Epstein–Barr virus (EBV) is frequently reactivated by coronavirus 2019 (COVID-19), and a high incidence of EBV viremia has been reported in patients with severe COVID-19. However, the impact of EBV viremia on progression to severe COVID-19 is unclear. Therefore, we conducted a study to evaluate the effect of EBV on COVID-19 progression.

We investigated EBV viremia at the time of admission in COVID-19 patients hospitalized between February 1, 2020, and April 11, 2021. A cross-sectional study was performed to compare the severity of COVID-19 according to the presence or absence of EBV viremia. However, since it is difficult to analyze the influence of EBV viremia on COVID-19 progression with cross-sectional studies, a retrospective cohort study, limited to patients with mild COVID-19, was additionally conducted to observe progression to severe COVID-19 according to the presence or absence of EBV viremia.

Two hundred sixty-nine COVID-19 patients were tested for EBV viremia. In a cross-sectional study that included patients with both mild and severe COVID-19, the EBV viremia group had more severe pneumonia than the EBV-negative group. However, in the cohort study limited to mild cases (N = 213), EBV viremia was not associated with COVID-19 progression.

COVID-19 severity may affect EBV viremia; however, there was no evidence that EBV viremia was a factor in exacerbating pneumonia in patients with mild COVID-19.

## 1 Introduction

In December 2019, a novel viral pneumonia was first reported in Wuhan, China,^[1]^ and the pathogen responsible was named severe acute respiratory syndrome coronavirus 2 (SARS-CoV-2).^[2]^ Coronavirus disease 2019 (COVID-19) spread worldwide from China and was detected in almost all countries by March 2020.^[3]^ COVID-19 is not only rapidly transmitted but is also fatal in patients with advanced and underlying disease.^[4]^

The effects of SARS-CoV-2 infection on immune function and the resultant reactivation of latent viruses are still under investigation. In 1 Italian study, Epstein–Barr virus (EBV) viremia was observed in 40 of 42 patients with severe COVID-19 and in 51 of 61 patients with severe COVID-19.^[5]^ It was also reported that patients with severe COVID-19 had higher levels of EBV viremia than those with mild COVID-19. However, the study was cross-sectional and did not reveal a causative relationship between severity and COVID-19. In particular, in most patients with severe COVID-19 at admission, several days had elapsed from symptom onset, so it was difficult to determine the causal relationship between EBV viremia and COVID-19 severity. Therefore, we conducted not only a cross-sectional study to analyze differences in COVID-19 severity depending on the presence or absence of EBV viremia, but we also conducted a retrospective cohort study, limited to patients with mild COVID-19, to investigate whether EBV viremia affects progression to severe COVID-19.

## 2 Methods

### 2.1 Study population

We conducted real-time polymerase chain reaction (PCR) assays to detect EBV in adult COVID-19 patients who were admitted to Inha University Hospital from February 1, 2020, through April 11, 2021. EBV PCR was routinely carried out at the time of admission, and if the test was not performed within 5 days, the patient was excluded from the study. Children (under 15 years old) were excluded from the study.

### 2.2 COVID-19 severity classification

COVID-19 severity was classified according to the following 6-grade system: Grade 1, symptomatic but no oxygen therapy required; Grade 2, low-flow nasal cannula oxygenation; Grade 3, high-flow nasal cannula/non-invasive ventilation; Grade 4, mechanical ventilation; Grade 5, extracorporeal membrane oxygenation; Grade 6, death.

### 2.3 Cross-sectional study

At the time of EBV viremia testing, we compared the COVID-19 severity of the EBV viremia group and the EBV-negative group. Specifically, between the groups, we compared the proportion of patients requiring oxygen therapy (Grade 2 or higher) and the proportion requiring at least high-flow nasal cannula ventilation (Grade 3 or higher). Lymphocyte subsets of blood sample obtained from patients were also compared between the EBV viremia group and the EBV-negative group.

### 2.4 Retrospective cohort study

Patients with Grade 2 COVID-19 or higher at the time of admission are often >1 week from disease onset. Therefore, in patients with at least Grade 2 disease, it is difficult to analyze whether EBV viremia is the cause or consequence of severe COVID-19. Therefore, we conducted a retrospective cohort study limited to patients with mild COVID-19 (Grade 1) at the time of hospitalization. Severity at the time of admission was defined as the worst grade within 24 hours of admission. Patients with mild COVID-19 at admission were divided into an EBV viremia group and an EBV-negative group, and progression to severe COVID-19 was observed. The grade of COVID-19 progression was defined as the worst grade within 60 days of admission or until discharge. The primary outcome was the need for oxygen therapy (Grade 2 or higher). The secondary outcome was the need for high-flow nasal cannula oxygenation (Grade 3 or higher).

### 2.5 COVID-19, EBV PCR test, and lymphocyte subpopulation analyses

For COVID-19 diagnoses, the Allplex 2019-nCoV Assay kit (Seegene Inc., Seoul, Republic of Korea) was used for PCR of upper or lower respiratory tract secretions. The Real-Q EBV Quantification Kit (BioSewoom, Inc., Seoul, Republic of Korea) was used to detect the EBV virus. The cut-off for EBV viremia was 72 copies/mL, which was the reference value given in the manufacturer's insert.

The lymphocyte subpopulation was analyzed using multicolor flow cytometry (BD FACSCanto II, Becton Dickinson, San Jose, CA). Whole blood samples were stained with BD Multitest CD3 FITC/CD8 PE/CD45 PerCP/CD4 APC and BD Multitest CD3 FITC/CD16+CD56 PE/CD45 PerCP/CD19 (Becton Dickinson, San Jose, CA) and analyzed according to the manufacturer's instructions. Each lymphocyte subpopulation was presented as an absolute count (cells/μL).

### 2.6 Statistical analysis

For the intergroup COVID-19 severity and lymphocyte subset comparisons, Fisher exact test and the Mann–Whitney *U* test were used. Logistic regression analysis (enter method) was used to analyze risk factors for progression to severe COVID-19. All tests were 2-tailed, and a *P*-value of .05 was considered statistically significant. Data analyses were performed using SPSS Statistics for Windows, version 21 (IBM Corp., Armonk, NY).

### 2.7 Ethics statement

This study was approved by the institutional review board of Inha University Hospital, Incheon, Republic of Korea. All patient records were anonymized.

## 3 Results

### 3.1 General characteristics of the COVID-19 group

During the study period, 359 adult patients diagnosed with COVID-19 were admitted to our hospital. Tests were performed for patients admitted to the general ward. Patients admitted directly to the intensive care unit (n = 29) were not tested and were excluded from the analysis. Patients not designated for EBV PCR testing (n = 53) were excluded. Patients who were not tested within 5 days after hospitalization (n = 8) were excluded. Finally, 269 patients were included in the COVID-19 group. The mean age of the patients was 61.6 years, and 59.5% were women. The median interval from hospitalization to EBV testing was 2.3 days. EBV viremia was found in 16.7% of COVID-19 patients, and the highest incidence (32.6%) was found in patients aged 70 to 79 (Table 1). According to grade at the time of hospitalization, EBV viremia incidence values were as follows: 30/211 (14.2%) for Grade1, 8/44 (18.2%) for Grade 2, 4/10 (40.0%) for Grade 3, 2/2 (100.0%) for Grade 4, and 1/1 (100.0%) for Grade 5 (Table 1).

**Table 1:** General characteristics and EBV viremia among COVID-19 patients.

Age group, yr	Severity at admission						Total, n	EBV viremia, n (%)
	Gr1, n (%)	Gr2	Gr3	Gr4	Gr5	Gr6		
15–29	18 (100.0)		–	–	–	–	18	2 (11.1)
30–39	35 (97.2)	1 (2.8)	–	–	–	–	36	0 (0.0)
40–49	25 (89.3)	2 (7.1)	1 (3.6)	–	–	–	28	2 (7.1)
50–59	27 (75.0)	7 (19.4)	1 (2.8)	–	1 (2.8)	–	36	7 (19.4)
60–69	34 (75.6)	8 (17.8)	3 (6.7)	–	–	–	45	5 (11.1)
70–79	28 (60.9)	13 (28.3)	3 (6.5)	2 (4.3)	–	–	46	15 (32.6)
≥80	45 (75.0)	13 (21.7)	2 (3.3)	–	–	–	60	14 (23.3)
Total	212 (78.8)	44 (16.4)	10 (3.7)	2 (0.7)	1 (0.4)	–	269	45 (16.7)

EBV = Epstein–Barr virus; Gr1, Grade 1 = symptomatic but no oxygen therapy required, Gr2, Grade 2 = low-flow nasal cannula, Gr3, Grade 3 = high-flow nasal cannula/non-invasive ventilation, Gr4, Grade 4 = mechanical ventilation, Gr5, Grade 5 = extracorporeal membrane oxygenation, Gr6, Grade 6 = death.

### 3.2 Cross-sectional study at the time of EBV viremia testing

At the time of blood EBV testing, the EBV-positive group had a high incidence of severe COVID-19 (15/45, 33.3%) compared with the EBV-negative group (42/224, 18.75%) (*P* = .04). Severe COVID-19 also occurred more frequently in the EBV-positive group (7/45, 15.6%) than the EBV-negative group (7/224, 3.1%) (*P* = .003, Table 2). There was no statistically significant difference in terms of lymphocyte subsets between the EBV-positive and EBV-negative groups (Table, Supplemental Digital Content). The mean CCI of the group without EBV viremia was 2.33 (SD 2.15) and the group with EBV viremia was 3.36 (SD 1.84), which indicates there was a statistically significant difference between the 2 groups (*P* = .001).

**Table 2:** Cross-sectional comparison of severity between the EBV-positive and EBV-negative groups.

	EBV-positive (n = 45)	EBV-negative (n = 224)	*P*-value
COVID-19 severity	n (%)	n (%)	
Gr1	30 (66.7%)	182 (81.3%)	
Gr2	8 (17.8%)	36 (16.7%)	
Gr3	4 (8.9%)	6 (2.7%)	
Gr4	2 (4.4%)	0 (0.0%)	
Gr5	1 (2.2%)	0 (0.0%)	
Gr6	0 (0.0%)	0 (0.0%)	
Moderate-severe (Gr2–6)	15 (33.3%)	42 (18.8%)	.04∗
Severe (Gr3–6)	7 (15.6%)	7 (3.1%)	.003∗

EBV = Epstein–Barr virus, Gr1, Grade 1 = symptomatic but no oxygen therapy required, Gr2, Grade 2 = low-flow nasal cannula, Gr3, Grade 3 = high-flow nasal cannula/non-invasive ventilation, Gr4, Grade 4 = mechanical ventilation, Gr5, Grade 5 = extracorporeal membrane oxygenation, Gr6, Grade 6 = death.

### 3.3 Retrospective cohort study among patients with mild-COVID-19

At the time of hospitalization, 213 people with mild COVID-19 were divided into 2 groups by the presence or absence of EBV viremia, and the groups’ progress was observed for 2 months or until discharge/death, whichever came first. Unlike the cross-sectional study, in the cohort study limited to mild cases, the incidence of progression to moderate or severe COVID-19 did not significantly differ between the 2 groups. Progression to severe COVID-19 was only found in the EBV-negative group (Table 3). Logistic regression analysis revealed age as a risk factor for progression to severe COVID-19; EBV infection was not identified as a risk factor for such a progression (Table 4).

**Table 3:** Retrospective cohort comparison of progression to severe COVID-19 between the EBV-positive and EBV-negative groups.

		EBV-negative (n = 224)	*P*-value
COVID-19 severity	COVID-19 severity	n (%)	
Gr1	Gr1	182 (81.3%)	
Gr2	Gr2	36 (16.7%)	
Gr3	Gr3	6 (2.7%)	
Gr4	Gr4	0 (0.0%)	
Gr5	Gr5	0 (0.0%)	
Gr6	Gr6	0 (0.0%)	
Moderate-severe (Gr2–6)	Moderate-severe (Gr2–6)	42 (18.8%)	.04∗
Severe (Gr3–6)	Severe (Gr3–6)	7 (3.1%)	.003∗

EBV = Epstein–Barr virus, Gr1, Grade 1 = symptomatic but no oxygen therapy required, Gr2, Grade 2 = low-flow nasal cannula, Gr3, Grade 3 = high-flow nasal cannula/non-invasive ventilation, Gr4, Grade 4 = mechanical ventilation, Gr5, Grade 5 = extracorporeal membrane oxygenation, Gr6, Grade 6 = death.

**Table 4:** Logistic regression analyses for progression to severe COVID-19.

Variable (N = 239)	No progression (Grade 1)	Progression (Grade 2–6)	Unadjusted			Adjusted		
			Unadjusted OR	95% CI	*P*-value	Adjusted OR	95% CI	*P*-value
Age								
<60	96	9	4.551	2.047–10.117	.001∗	3.801	1.545–9.350	.004∗
≥60	75	32						
Sex								
Female	106	26	0.941	0.464–1.907	.866	0.913	0.408–2.044	.824
Male	65	15						
History of MI								
No	170	40	4.250	0.260–69.411	.310	4.626	0.255–54.084	.301
Yes	1	1						
Congestive heart failure								
No	171	41	–	–	–	–	–	–
Yes	0	0						
PAOD								
No	171	41	–	–	–	–	–	–
Yes	0	0						
History of CVA								
No	165	35	4.714	1.436–15.479	.011∗	3.906	0.862–17.703	.077
Yes	6	6						
Dementia								
No	159	34	2.728	1.001–7.438	.050∗	1.861	0.585–5.924	.293
Yes	12	7						
COPD								
No	170	40	4.250	0.260–69.411	.310	1.797	0.021–152.183	.796
Yes	1	1						
Connective tissue diseases								
No	168	41	–	–	–	–	–	–
Yes	3	0						
Peptic ulcer disease								
No	169	41	–	–	–	–	–	–
Yes	2	0						
Chronic liver diseases								
No	169	41	–	–	–	–	–	–
Yes	2	0						
Diabetics mellitus								
No	147	27	3.176	1.461–6.904	.004∗	2.029	0.861–4.782	.106
Yes	24	14						
Hemiplegia								
No	168	39	2.872	0.464–17.774	.257	0.691	0.228–2.097	.515
Yes	3	2						
Chronic kidney diseases								
No	169	40	2.112	0.187–23.878	.546	1.548	0.484–6.391	.391
Yes	2	1						
Solid organ tumor								
No	167	39	2.141	0.379–12.111	.389	1.548	0.183–13.090	.688
Yes	4	2						
Leukemia								
No	171	41	–	–	–	–	–	–
Yes	0	0						
Lymphoma								
No	171	41	–	–	–	–	–	–
Yes	0	0						
AIDS								
No	171	41	–	–	–	–	–	–
Yes	0	0						
EBV viremia								
**No**	145	37	0.603	0.198–1.835	.373	0.340	0.099–1.164	.086
Yes	26	4						

CI = confidence interval, COPD = chronic obstructive pulmonary disease, CVA = cerebrovascular accident, EBV = Epstein–Barr virus, Grade 1 = symptomatic but no oxygen therapy required, Grade 2 = low-flow nasal cannula, Grade 3 = high-flow nasal cannula/non-invasive ventilation, Grade 4 = mechanical ventilation, Grade 5 = extracorporeal membrane oxygenation, Grade 6 = death, MI = myocardial infarction, OR = odds ratio, PAOD = peripheral arterial occlusive disease.

## 4 Discussion

EBV is latent in near 90% of people, which is the highest rate among herpes viruses.^[6]^ In patients with severe COVID-19, reactivation of viruses, such as herpes simplex, CMV, and EBV, occurs, and functional exhaustion of cytotoxic lymphocytes has been suggested as the cause.^[7,8]^ COVID-19 can cause cellular immune dysfunction^[8]^; therefore, it can induce reactivation of latent viruses. Several studies have reported a high incidence of reactivated EBV in COVID-19 patients.^[5,10]^ Additionally, COVID-19 has been reported to be more severe in patients with EBV viremia. However, this evidence was derived from cross-sectional studies; therefore, it is not known whether EBV viremia affected the progression in COVID-19 severity. The studies mainly investigated severely ill patients, and no intervals to testing were reported; therefore, the effect of EBV viremia on progression to severe COVID-19 may have been overestimated. However, in the present study, we performed the tests within a median of 2.3 days, and we conducted a cohort study limited to patients with mild COVID-19; thus, we mitigated the possibility of selection bias in favor of critical illness.

To observe the effects of EBV viremia, we conducted a cohort study to compare the COVID-19—associated acute respiratory distress syndrome progression in the EBV viremia and EBV-negative groups at the time of hospital admission. Although the incidence of EBV viremia varied by COVID-19 severity at admission, there was not a higher probability of progression in severity in the EBV viremia group. Although the number of events was small, the incidence of progression was low in the EBV viremia group; at least early EBV viremia does not seem to affect COVID-19 prognosis. EBV viremia is common, even in patients severely ill with diseases other than COVID-19. One study reported that EBV DNA is detected in the lower respiratory tract of patients with severe respiratory tract infections in which no other pathogen has been detected.^[11]^ It is difficult to conclude that EBV has a cytopathic effect in all cases where other pathogens have not been identified because it is not easy to identify the causative pathogen of pneumonia in many cases. Still, some researchers claim reactivated EBV may be pathogenic as a result of a compromised immune system, whereas others claim that it is just an indicator of severe illness.^[12]^

However, for severe COVID-19, the impact of viremia may be different from that of the present study. For patients with severe COVID-19, steroid administration is often prolonged. Also, host immunity may be compromised due to critical illness. In these cases, EBV viremia can persist at high levels. During EBV reactivation, EBV can interfere with the activity of natural killer cells and helper T cells.^[12]^ EBV causes B-cell transformation^[13]^ and produces proteins that primarily impair interferon production during the lytic phase.^[14]^ Via this mechanism, EBV infection or reactivation can impair defenses to infection by other pathogens. This persistent viremia can reduce immunity for the reasons mentioned above, and this immunocompromised status can become part of a vicious cycle that worsens EBV viremia. Therefore, in severely ill patients, including those undergoing long-term steroid treatment, further investigations of EBV viremia may be needed.

This study had some limitations. First, there was no follow-up test for EBV viremia; therefore, this study could not confirm that EBV viremia persisted in severely ill patients. Second, the study included a relatively small number of patients. Additional studies with larger sample sizes are needed, and the mechanism of EBV viremia must be determined. However, even accounting for this, there is no evidence that early EBV viremia causes severe COVID-19. Third, there was a statistically significant difference in mean CCI between the group with and without EBV viremia. However, since the CCI of the group with EBV was higher than that of the group without EBV viremia, if the CCI is adjusted, the prognosis of the group with EBV viremia is likely to be better than our suggested value. Therefore, it seems the difference in CCI between the 2 groups hardly changes our conclusion.^[9]^

## Author contributions

**Conceptualization:** Jae Hyoung Im, Mi Hwa Park.

**Data curation:** Young Soo Je, Ji-Hun Jang, Jung Soo Kim, Jun Hyeok Lim.

**Formal analysis:** Chung Hyun Nahm.

**Supervision:** Mi Hwa Park.

**Writing – original draft:** Jae Hyoung Im, Mi Hwa Park.

**Writing – review & editing:** Jin-Soo Lee, Ji Hyeon Baek, Hea Yoon Kwon, Moon-Hyun Chung, Mi Hwa Park.

## Supplementary Material

Supplemental Digital Content
