# Supplementary material for: The effect of Epstein–Barr virus viremia on the progression to severe COVID-19
Source: Medicine (Baltimore). 2022 May 13;101(18):e29027. doi: 10.1097/MD.0000000000029027 (PMC9276369; doi:10.1097/MD.0000000000029027)
Supplement: Supplemental Digital Content [file medi-101-e29027-s001.doc]

| **Supplementary Table 1. Comparison of lymphocyte subsets between EBV-positive and EBV-negative groups** | | | |
| --- | --- | --- | --- |
|  |  |  |  |
| **Lymphocyte subset** | **EBV–positive (n=45)** | **EBV–negative (n=224)** | ***p*-value** |
| CD3 [IQR] | 0.84 [0.57–1.09] | 0.67 [0.46–1.04] | 0.121 |
| CD4 [IQR] | 0.47 [0.35–0.74] | 0.41 [0.27–0.61] | 0.069 |
| CD8 [IQR] | 0.33 [0.16–0.44] | 0.28 [0.16–0.42] | 0.466 |
| CD19 [IQR] | 0.13 [0.08–0.25] | 0.11 [0.08–0.18] | 0.232 |
| CD56 [IQR] | 0.21 [0.11–0.38] | 0.18 [0.12–0.28] | 0.319 |
|  |  |  |  |
| EBV, Epstein–Barr virus; IQR, interquartile range | | | |
